# A case of toxic epidermal necrolysis after cabotegravir initiation

**DOI:** 10.1016/j.jdcr.2026.08.035

**Published:** 2026-08-28

**Authors:** Rhea Bhargava, Timothy O'Connor, Margaret E. Scollan, Andres Arredondo Santana, Alejandro Gru, Barbara Magid

**Affiliations:** a Department of Emergency Medicine, New York-Presbyterian Columbia-Cornell, New York, New York; b Department of Dermatology, New York-Presbyterian Columbia, New York, New York; c Department of Emergency Medicine, Columbia University Vagelos College of Physicians and Surgeons, Manhattan, New York; d Department of Dermatology, Columbia University Vagelos College of Physicians and Surgeons, Manhattan, New York

**Keywords:** cabotegravir, human immunodeficiency virus, preexposure prophylaxis, Stevens-Johnson syndrome, toxic epidermal necrolysis

## Introduction

Human immunodeficiency virus affects millions of people in the United States. Several medications exist for human immunodeficiency virus preexposure prophylaxis (PrEP), ranging from daily pills to long-acting injectables. Cabotegravir (brand name Apretude*,* manufactured by ViiV Healthcare) is a Food and Drug Administration−approved long-acting injection used for PrEP, which is generally well tolerated with mild adverse effects. Herein, we report a case of toxic epidermal necrolysis (TEN) in a 30-year-old man who had recently initiated PrEP with cabotegravir. Stevens-Johnson syndrome (SJS)/TEN represents a spectrum of severe, T-cell-mediated, drug-induced skin eruptions, with severity and mortality increasing with the extent of cutaneous involvement (up to 10% in SJS and 50% in TEN).^1^^,^^2^

Cabotegravir prevents human immunodeficiency virus by inhibiting integrase, thus blocking viral DNA integration into host cells. The formulation of cabotegravir allows for dosing every 8 weeks after an initial period of monthly injections, maintaining protective drug levels and improving adherence compared with daily oral PrEP.^3^ Cabotegravir is generally well tolerated, but common adverse reactions include injection site reactions, fatigue, gastrointestinal upset, depression, and, rarely, severe hypersensitivity reactions.^4^ However, there have not been any known case reports to date associating cabotegravir with SJS/TEN.^5^^,^^6^

## Case report

A 30-year-old man with a remote history of treated syphilis presented to the emergency department endorsing fevers, malaise, periorbital edema, and a new painful rash along his arms, chest, and back. He had initiated cabotegravir 11 to 12 days prior to development of fevers and malaise. Approximately 24 to 48 hours after the initial fever, the patient began to develop a rash. He denied recent illness or other new medication exposures in the past 6 months. The patient was tachycardic with extensive mucositis, bilateral conjunctival injection, and progressing erythematous desquamative papules and plaques on his scalp, face, trunk, and extremities with a positive Nikolsky sign (Fig 1).

**Fig 1:**
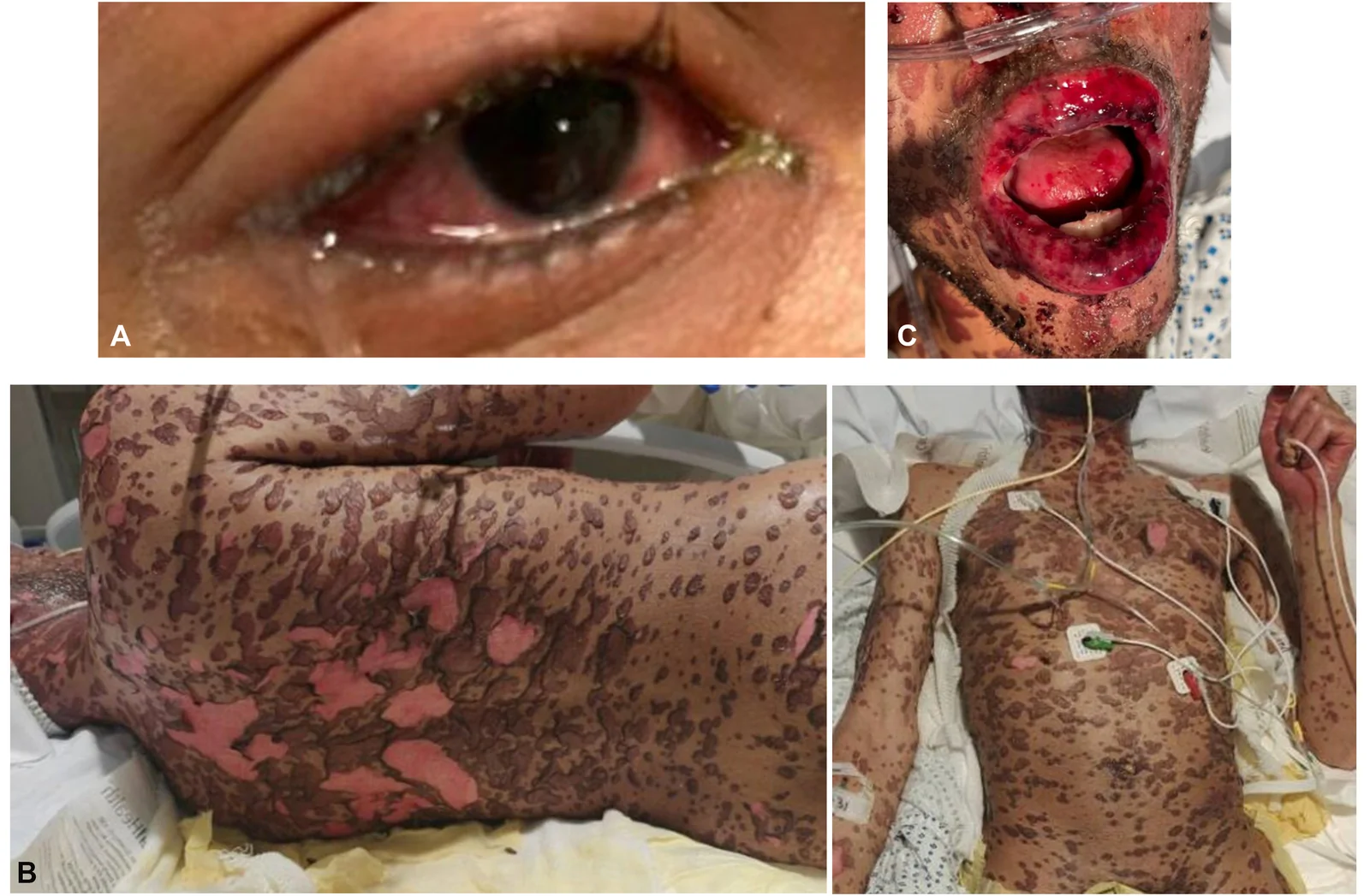
Patient’s ocular, cutaneous, and mucosal findings, including **(A)** conjunctival injection, **(B)** diffusely scattered sloughing bullae and desquamation of the trunk and extremities, and **(C)** labial and lingual erosion.

Laboratory workup was notable for mild leukocytosis with normal absolute eosinophils of 0.1%, elevated C-reactive protein, and lactic acidosis. Epstein-Barr virus, respiratory pathogen panel, chlamydia/gonorrhea, monkeypox and orthopoxvirus polymerase chain reaction, hepatitis B virus viral load, and herpes simplex polymerase chain reaction were negative. Mycoplasma immunoglobulin M was positive.

TEN was the leading differential diagnosis. The patient lacked clusters of micropustules in intertriginous areas, as are seen in acute generalized exanthematous pustulosis, nor did he have annular plaques bordered by rows of single vesicles as in linear IgA bullous dermatosis. Additionally, he did not present with eosinophilia, transaminitis, and lymphadenopathy characteristic of drug reaction with eosinophilia and systemic symptoms. A punch biopsy of the skin demonstrated epidermal necrosis and apoptosis, basal layer vacuolization with subepidermal blistering, confirming a diagnosis of TEN (Fig 2). Direct immunofluorescence was not collected given lower suspicion for other etiologies. The algorithm of drug causality for epidermal necrolysis was 3, corresponding to a “possible” causal link, suggesting that the implicated drug could be cabotegravir.^1^

**Fig 2:**
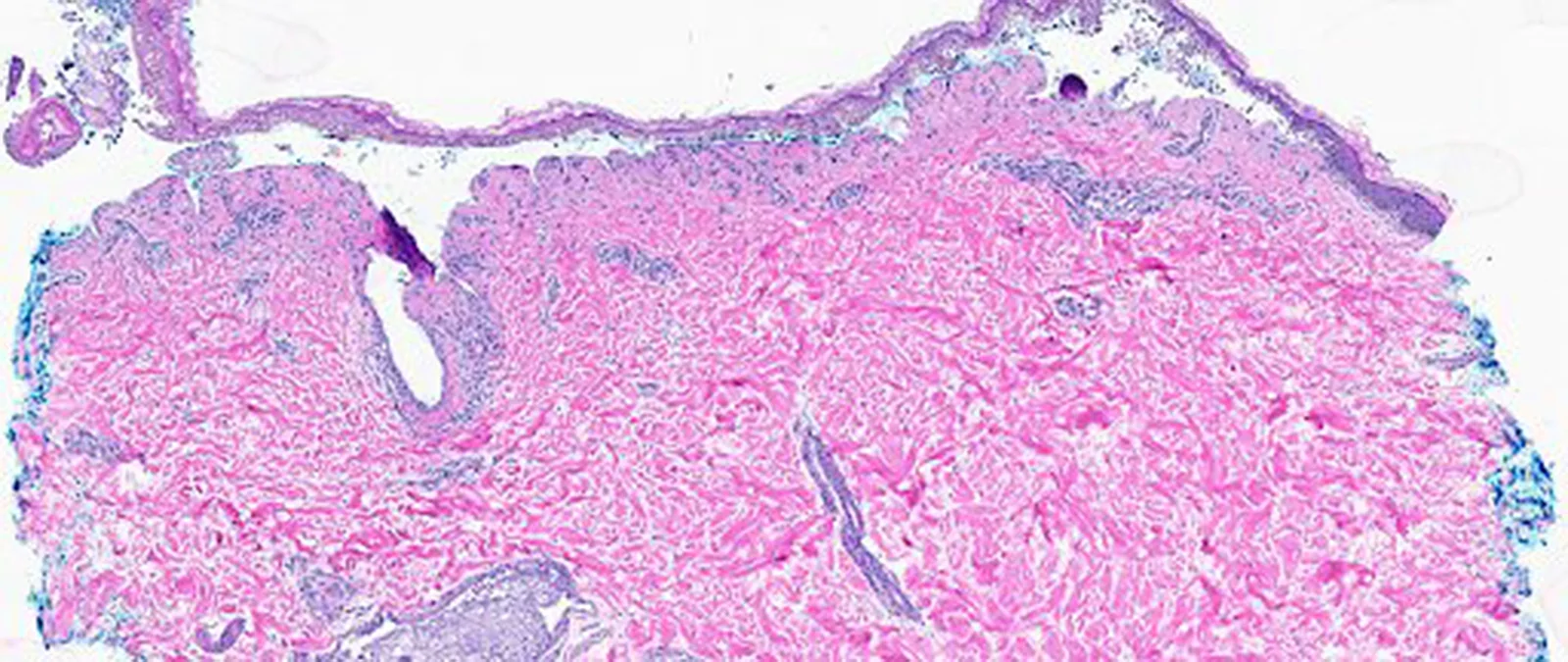
Histopathology demonstrating full-thickness epidermal necrosis and apoptosis, basal layer vacuolization with a subepidermal blister, and a superficial perivascular lymphocytic infiltrate compatible with TEN.

The severity-of-illness score for TEN was calculated as 2, indicating an estimated mortality rate of 12.1% (Fig 3).^7^

**Fig 3:**
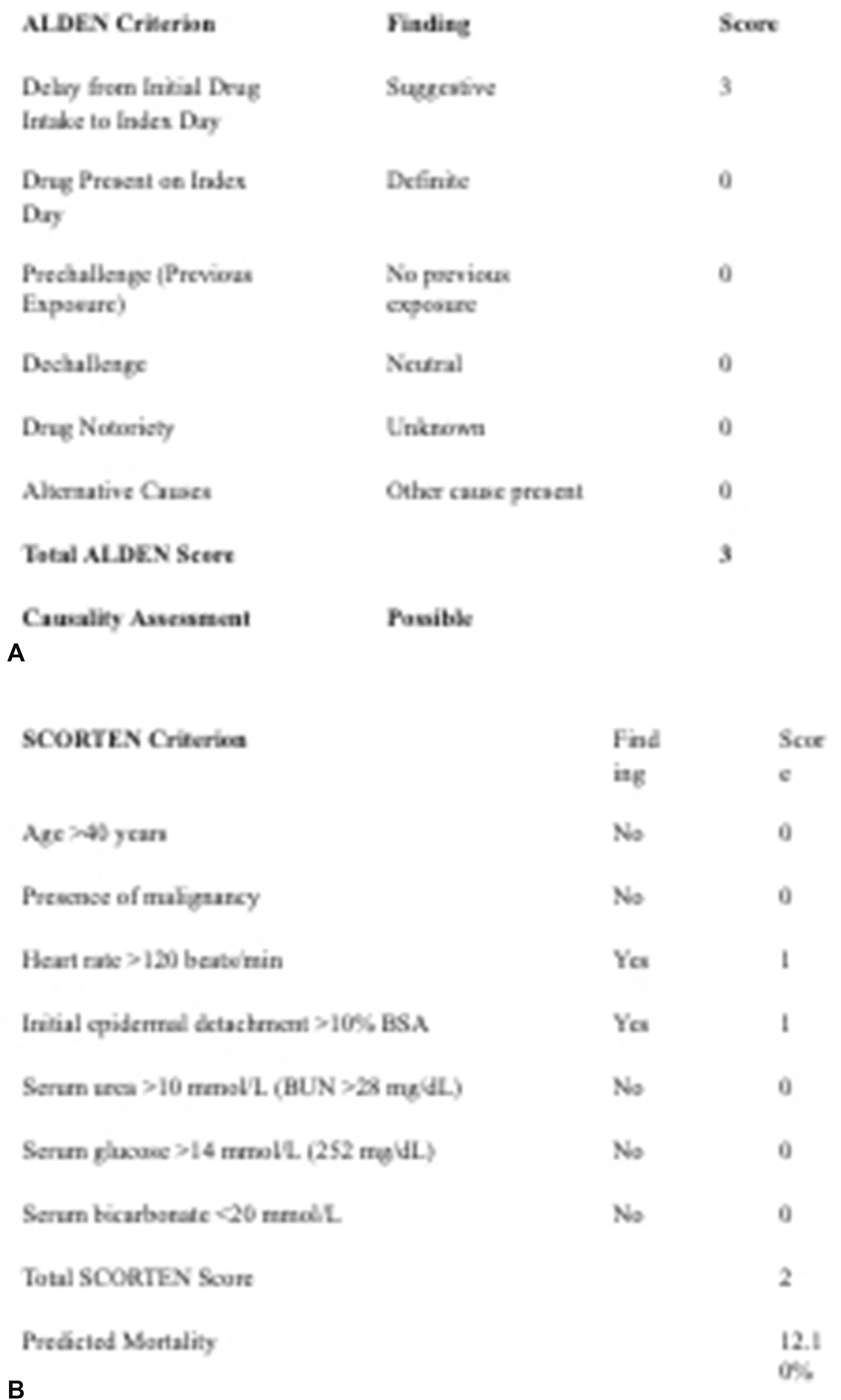
**A,** Algorithm of drug causality for epidermal necrolysis (ALDEN) score of 3, indicating possible causality^1^. **B,** Severity-of-illness score for TEN (SCORTEN) score of 2, for heart rate >120 beats per minute and initial epidermal detachment >10% body surface area, indicating 12.1% mortality.^7^

The patient’s cutaneous symptoms progressed rapidly, eventually involving >30% body surface area, so the decision was made to transfer to an outside intensive care unit with burn capabilities. In addition to extensive wound care and pain management, the patient ultimately received a total of 6 doses of etanercept, along with 3 days of intravenous immunoglobulin. While typical dosing of etanercept for SJS/TEN is 2 doses 72 hours apart, given the patient's disease severity, it was determined that the benefit of additional doses outweighed the risk. Although the overall effectiveness of intravenous immunoglobulin in the treatment of TEN remains controversial, some studies suggest that initiating intravenous immunoglobulin within 7 days of rash onset can significantly reduce lesion progression by stopping epidermal necrosis.^8^ The patient was discharged in good condition after a 43-day hospital stay.

## Discussion

Although PrEP medications are overall well-tolerated, there have been rare reports of severe cutaneous adverse reactions to their components, including tenofovir^9^ (a component of Truvada and Descovy), and this case represents the first known report of TEN following cabotegravir initiation. TEN represents the most life-threatening end of the severe cutaneous adverse reaction spectrum, and outcomes have been shown to correlate with medication half-life. In an observational study of 113 patients with SJS/TEN, exposure to causative drugs with a long half-life elimination (defined in this study as >24 hours) is associated with a markedly increased risk of death compared with drugs with shorter half-lives. This suggests that prolonged presence of an offending agent exacerbates tissue damage and systemic inflammation.^1^ The first step in SJS/TEN treatment involves discontinuing the culprit drug; however, cabotegravir is an injectable formulation with a half-life of up to 12 weeks, making its abrupt elimination difficult.^3^^,^^10^ Given cabotegravir is a widely prescribed medication for PrEP, this case suggests that further research is warranted to better characterize the risk of SJS/TEN associated with drug initiation.

## Limitations

Although the patient denied recent illnesses, a subclinical viral etiology cannot be excluded. Mycoplasma immunoglobulin M was positive; however, immunoglobulin M levels can remain positive for weeks to years after an infection, and positivity does not necessarily indicate active disease. The possibility of a reaction to excipients (polysorbate 20, mannitol) cannot be excluded, although this is exceedingly rare. Additionally, given the patient’s severe, life-threatening presentation requiring months-long hospitalization, it would be unexpected for a subclinical virus to have caused such a severe presentation. Another limitation of this study is the lack of human leukocyte antigen genotype analysis. Various human leukocyte antigen genotypes have been found to correlate with increased sensitivity to specific drugs, such as human leukocyte antigen-B∗1502 and carbamazepine. This case could have helped establish such a possible correlation with cabotegravir; however, no such analysis was performed. Additionally, although cabotegravir has a known half-life, actual drug and metabolite concentrations were never measured. Despite this, and although further workup is warranted, we believe that our study provides a strong evidence base in establishing TEN as a rare but serious complication of cabotegravir.

## Conflicts of interest

None disclosed.
